# Surgical management of intraventricular hemorrhage and posthemorrhagic hydrocephalus in premature infants – single center experience

**DOI:** 10.3389/fped.2025.1610697

**Published:** 2025-08-07

**Authors:** David Krahulik, Martin Wita, Vanda Volkova, Jan Halek, Jan Krahulik, Filip Blazek

**Affiliations:** ^1^ Department of Neurosurgery, University Hospital Olomouc, Olomouc, Czechia; ^2^ Department of Neonatology, University Hospital Olomouc, Olomouc, Czechia; ^3^ Department of Neurosurgery, University Hospital Ostrava, Ostrava, Czechia

**Keywords:** intraventricular hemorrhage, newborns, hydrocephalus, neonatal neurosurgery, premature infants

## Abstract

**Introduction:**

Intraventricular hemorrhage (IVH) is a severe complication of prematurity, often leading to posthemorrhagic hydrocephalus (PHH). While advances in neonatal care have reduced the incidence of IVH, managing progressive PHH remains challenging. Surgical interventions are crucial for reducing intracranial pressure and preventing long-term neurodevelopmental deficits. This study presents our single-center experience in the surgical management of IVH and PHH in premature infants, highlighting outcomes associated with different surgical strategies.

**Methods:**

This retrospective study was conducted at a tertiary neonatal care center between 2017 and 2023. Premature infants (≤32 weeks of gestation or <1,500 g birth weight) were screened for IVH using cranial ultrasound. IVH was graded using the modified Papile classification. Clinical data, imaging findings, and surgical outcomes were collected. Temporizing measures included ventricular access devices (VAD), ventriculosubgaleal (VSG) shunts, and external ventricular drainage (EVD), with permanent ventriculoperitoneal (VP) shunts used for long-term management. Neurodevelopmental outcomes were evaluated at one-year follow-up.

**Results:**

The study included 402 premature infants, of whom 75 (18.7%) were diagnosed with IVH. Among these, 6 infants developed PHH requiring surgical intervention. VAD shunting was the preferred temporizing measure due to lower infection rates, with an average duration of 35 days before conversion to VP shunting in 4 children. At one-year follow-up, 64% of infants demonstrated no significant neurological impairment, while 23.2% had severe complications, including developmental delays or motor deficits. Mortality was 20.1%, primarily in infants with Grade III-IV IVH. Logistic regression analysis identified gestational age, corticosteroid treatment, and inborn status as significant predictors of favorable outcomes.

**Conclusion:**

Effective surgical management of PHH in premature infants hinges on timely diagnosis, early intervention, and appropriate selection of temporizing measures. Ventricular access devices (VAD) offer a safe and effective strategy to stabilize infants before permanent shunt placement, minimizing complications and improving long-term outcomes. Close collaboration between neonatologists and neurosurgeons remains essential to optimizing care and reducing morbidity in this high-risk population.

## Introduction

Intraventricular hemorrhage (IVH) is a significant complication of prematurity, contributing to increased morbidity and mortality in neonates. It primarily originates from the germinal matrix, a highly vascularized area in the developing brain, particularly susceptible to hemorrhage due to its unique anatomic and vascular characteristics (1). The germinal matrix's fragile vessels, lacking muscular and collagenous support, combined with the immature autoregulatory mechanisms in preterm infants, predispose them to IVH. IVH typically occurs within the first 72 h of life, with catastrophic, saltatory, or clinically silent presentations. Advances in neonatal care have reduced the incidence of IVH, yet posthemorrhagic hydrocephalus (PHH) remains a formidable challenge for pediatric neurosurgeons (2).

The pathogenesis of PHH is multifaceted, involving the obstruction of cerebrospinal fluid (CSF) pathways due to blood clots, leading to ventricular dilation and subsequent white matter injury from the release of free iron and other neurotoxic substances. This condition is particularly prevalent among infants with a birth weight of less than 1,500 g and those born before 32 weeks of gestation. Among preterm infants with IVH, 20%–50% develop ventriculomegaly, which can be transient or progressive, necessitating timely and effective intervention (3).

Management strategies for PHH in preterm infants are diverse, ranging from conservative measures to more invasive neurosurgical interventions. Pharmacological treatments aimed at reducing CSF production have not demonstrated significant benefit and may lead to adverse outcomes. Techniques such as drainage, irrigation, and fibrinolytic therapy (DRIFT) and neuroendoscopic lavage have shown promise in improving cognitive outcomes and reducing infection rates, although their adoption remains limited due to mixed results and the need for further multicenter validation (4).

Temporary CSF diversion methods, including serial lumbar punctures, ventriculosubgaleal shunts (VSGS), external ventricular drainage (EVD), and ventricular access devices (VAD), offer interim solutions while the infant gains sufficient weight and stability for permanent ventriculoperitoneal shunting. Each technique has its advantages and drawbacks (5). For instance, VSGS is preferred for its lower infection rates and simplicity, while VAD allows for controlled CSF aspiration and ease of conversion to a permanent shunt. However, these methods are not without complications, including catheter migration, infection, and subgaleal encephaloceles.

Early surgical intervention and the choice of temporizing measures play a crucial role in determining long-term outcomes. Studies have shown that temporizing strategies like VAD insertion before ventriculoperitoneal shunt (VP shunt) placement are associated with fewer revisions and lower morbidity compared to direct VP shunt insertion (6). The decision-making process for selecting the optimal treatment modality should be individualized, considering the infant's clinical condition, gestational age, and institutional capabilities.

Despite advancements in surgical techniques and neonatal intensive care, the management of PHH remains challenging. Long-term follow-up data indicate that lower gestational age and higher-grade IVH are associated with increased risks of shunt complications and neurodevelopmental impairments (7). The establishment of standardized guidelines and multicenter collaborations is essential to improve outcomes and reduce variability in care. As the understanding of PHH evolves, further research is warranted to refine surgical strategies and explore innovative treatment modalities, ultimately enhancing the prognosis for this vulnerable patient population.

## Methods

This retrospective study was conducted at a single tertiary neonatal care center at the University Hospital Olomouc between 2017 and 2023 to evaluate the clinical characteristics, surgical management, and outcomes of premature infants with and without intraventricular hemorrhage (IVH) and posthemorrhagic hydrocephalus (PHH). Institutional ethics approval was obtained prior to the study.

The study population consisted of all neonates born at ≤32 weeks of gestation or with a birth weight <1,500 g who underwent routine cranial ultrasound (cUS) screening during the neonatal period. Diagnosis and grading of IVH were based on the modified Papile classification (grades I–IV). No cases of antenatally diagnosed IVH were identified in our cohort; all diagnoses were made postnatally via routine cUS performed within the first 72 h of life.

Clinical data were collected across multiple categories, including gestational age, birth weight, gender, twin status, and inborn or outborn birth status. Perinatal data included APGAR scores at 5 min, the need for intubation in the delivery room, and the administration of antenatal corticosteroids.

The presence and timing of IVH were documented, with its first manifestation recorded by day of life. The laterality of IVH (unilateral vs. bilateral) was also noted. Posthemorrhagic ventricular dilation was monitored using ultrasound and MRI, measuring ventricular dimensions such as anterior horn width (AHW), ventricular index (VI) and thalamo-occipital distance (TOD) (Table 1). Maximal ventricular diameter before shunt implantation was recorded using various imaging modalities. The following thresholds were used to guide surgical intervention: anterior horn width (AHW) > 6 mm, ventricular index (VI) exceeding the 97th percentile for gestational age, and thalamo-occipital distance (TOD) > 25 mm. Intervention was considered when these thresholds were accompanied by signs of increased intracranial pressure such as bulging fontanelle, rapid head circumference growth, or apnea.

**Table 1:** Descriptive statistics of cranial ultrasound and MRI parameters in the study cohort.

Parameter	Mean	Standard deviation	Minimum	Percentile 25	Median	Percentile 75	Maximum	Count
GV	28.9	2.4	22.0	26.9	29.6	31.0	31.9	402.0
PH (gr.)	1,171.6	395.6	290.0	830.0	1,180.0	1,490.0	2,130.0	402.0
UPV	8.14	11.66	1.00	1.00	5.00	10.00	69.00	136.00
VI SIN	10.18	2.28	5.60	8.75	9.85	11.60	14.00	24.00
AHW SIN	5.75	4.26	0.90	2.35	4.25	8.70	15.30	24.00
VI DX	10.55	2.54	7.80	8.95	9.80	11.70	19.00	24.00
AHW DX	5.86	4.66	0.20	1.60	5.75	8.20	17.30	24.00
TOD SIN	9.23	9.90	0.00	0.70	6.95	15.75	27.00	8.00
TOD DX	10.48	11.95	0.00	0.60	4.95	21.35	30.00	8.00
VI SIN 2	14.98	2.15	12.00	13.50	15.45	16.45	17.00	4.00
AHW SIN2	12.48	5.67	8.00	8.65	10.65	16.30	20.60	4.00
VI DX2	14.75	1.64	12.80	13.40	15.00	16.10	16.20	4.00
AHW DX2	13.03	5.71	8.00	9.45	11.45	16.60	21.20	4.00
TOD SIN2	33.50	12.92	17.90	23.00	35.05	44.00	46.00	4.00
TOD DX2	29.55	10.02	16.70	21.60	31.75	37.50	38.00	4.00

Surgical management was tailored to the clinical condition and imaging findings. All patients with PHH were treated using ventricular access devices (VADs). Permanent ventriculoperitoneal (VP) shunts were placed in neonates with persistent hydrocephalus once they achieved clinical stability, had a body weight of more than 2,000 g, and a CSF protein level of less than 1.5 g/L. The stabilization process prior to shunt placement was documented, along with the type of initial and subsequent surgical interventions.

Outcome measures focused on both short- and long-term results. Mortality and survival outcomes were tracked throughout the neonatal period. Neurodevelopmental outcomes were evaluated at one year, focusing on motor and cognitive development and categorized into normal development, paresis, plegia, or delay in psychomotor development (PMD). The need for repeated CSF taps, the presence of a subgaleal port, and the progression to ventriculoperitoneal shunting were documented. Neurodevelopmental outcomes were assessed at one year by pediatric neurologists using structured clinical evaluations, including assessment of tone, motor milestones, and cognitive-behavioral development. Formal neurodevelopmental scales such as the Bayley Scales were not consistently applied across all patients.

Statistical analysis was performed using IBM SPSS Statistics Version 23.0. Continuous variables were assessed for normality using the Shapiro–Wilk test and summarized as means or medians, depending on distribution. Group comparisons were performed using the Mann–Whitney U test for continuous variables and Fisher's exact test for categorical data. Kaplan–Meier survival analysis was used to assess shunt survival, and the log-rank test was applied for survival curve comparisons. Logistic regression analysis was used to identify predictors of IVH, PHH, and shunt-related complications. A significance level of *p* < 0.05 was set for all statistical tests.

## Results

The cohort comprised 402 premature infants, with detailed analyses focusing on both quantitative and qualitative data to evaluate risk factors for intraventricular hemorrhage (IVH), mortality, and long-term outcomes. Quantitative data were described using means, medians, standard deviations (SD), and ranges. The Shapiro–Wilk test confirmed that most continuous variables did not follow a normal distribution, prompting the use of the Mann–Whitney U test for between-group comparisons. Categorical data were analyzed using Fisher's Exact Test.

The mean gestational age (GA) was 28.9 weeks (SD ± 2.4), and the median was 29.6 weeks. Birth weight ranged from 290 to 2,130 g, with a mean of 1,171.6 g. Most infants (76.2%) were born between 28 + 0 and 31 + 6 weeks. However, those born at 24 + 0 to 25 + 6 weeks had the highest incidence of severe IVH. Statistical analysis showed a strong association between lower GA and increased risk of severe IVH (*p* < 0.0001). Cranial ultrasound measurements indicated that anterior horn width (AHW) and thalamo-occipital dimension (TOD) were significant indicators of post-hemorrhagic hydrocephalus (PHH). The mean AHW in infants with PHH was 12.48 mm, exceeding diagnostic thresholds and correlating with clinical signs of elevated intracranial pressure.

The qualitative data provided insights into demographic and clinical characteristics. Of the infants analyzed, 58.2% were male, while 41.8% were female (Table 2). Twin pregnancies accounted for 18.9% of the cohort. Antenatal corticosteroid administration emerged as a crucial protective factor. Among the infants, 62.7% received complete corticosteroid courses, significantly reducing the risk of IVH (*p* = 0.004). In contrast, incomplete or absent corticosteroid treatment was associated with a higher incidence of severe IVH. Inborn status, defined as being born in a hospital with advanced neonatal care facilities, was another significant protective factor. Among the cohort, 93% of infants were inborn, and these infants had a markedly lower risk of IVH compared to outborn infants (*p* = 0.002) (Table 3). APGAR scores at five minutes indicated generally favorable neonatal conditions (Table 4), with 38.4% scoring 10 and 28.9% scoring 9. Lower scores were more common in infants who developed severe IVH.

**Table 2:** Demographic and clinical characteristics of the entire cohort.

Demographic and clinical characteristics			
Variable	Category	Count	%
Gender	Male	234	58.2%
	Female	168	41.8%
Twin	No	326	81.1%
	Yes	76	18.9%
Inborn	No	28	7.0%
	Yes	372	93.0%

**Table 3:** Clinical interventions and outcomes.

Clinical data and outcomes			
Variable	Category	Count	%
IVH (Intraventricular hemorrhage)	No	327	81.3%
	Yes	75	18.7%
Intubation in delivery room	No	334	83.1%
	Yes	68	16.9%
Corticosteroid treatment	None	56	14.0%
	Incomplete	93	23.3%
	Complete	250	62.7%
Hemodynamic instability	No	251	62.4%
	Yes	141	37.6%

**Table 4:** Summary statistics for key clinical variables.

Summary statistics							
Variable	Mean	SD	Min	P25	Median	P75	Max
APGAR at 5 min	8.6	1.9	0.0	8.0	9.0	10.0	10.0
Day of first manifestation	5.5	5.6	1.0	3.0	4.0	7.0	31.0

Logistic regression analysis identified several independent predictors for the development of IVH (Table 5). Gestational age and corticosteroid administration were the most significant protective factors. Each additional week of gestation reduced the odds of developing IVH by 26%. Infants born at 28 + 0 to 31 + 6 weeks had a markedly lower risk of IVH compared to those born at 24 + 0 to 25 + 6 weeks (OR = 0.132, *p* < 0.0001). Complete corticosteroid treatment reduced the odds of IVH by 78% (OR = 0.221, *p* < 0.0001), while incomplete corticosteroid treatment provided some protection but to a lesser extent (OR = 0.361, *p* = 0.018). Inborn status was associated with significantly reduced odds of IVH (*p* = 0.002).

**Table 5:** Multivariate logistic regression analysis identifying predictors of IVH development.

Dependent variable: IVH (intraventricular hemorrhage)				
Variable	Sig.	OR	95% C.I. for OR	
			Lower	Upper
GV group	<0.0001			
24 + 0–25 + 6 vs. 26 + 0–27 + 6	0.002	0.262	0.112	0.614
24 + 0–25 + 6 vs. 28 + 0–31 + 6	<0.0001	0.132	0.067	0.259
Corticosteroids	0.0003			
Not given - complete	0.0001	0.221	0.105	0.463
Not given - incomplete	0.018	0.361	0.155	0.839
Twins (no – yes)	0.015	0.320	0.128	0.801
Constant	<0.0001	0.297		

Analysis of IVH and mortality revealed that the combination of IVH and death was most frequent in infants born at 24 + 0 to 25 + 6 weeks, with 61.3% experiencing either severe IVH or death (Table 6). Mortality was significantly higher in infants with Grade III-IV IVH compared to those with mild or no IVH. Complete corticosteroid administration was associated with improved survival outcomes, significantly reducing mortality compared to infants who did not receive corticosteroids. Gender did not significantly influence mortality (*p* = 1.0), while inborn infants had significantly better survival rates compared to outborn infants (*p* = 0.018).

**Table 6:** Distribution of IVH severity and mortality.

Group		IVH or death								*p*-value (Fisher's exact test)
		No significant bleeding		IVH III.		IVH IV.		Death count		
		Count	Column *N* %	Count	Column *N* %	Count	%	Count	Column *N* %	
GV groups	24 + 0–25 + 6	29	8.4%	3	33.3%	5	71.4%	19	61.3%	<0.0001
	26 + 0–27 + 6	49	14.1%	1	11.1%	1	14.3%	9	29.0%	
	28 + 0–31 + 6	269	77.5%	5	55.6%	1	14.3%	3	9.7%	
Corticosteroids	Not given	40	11.6%	1	11.1%	5	71.4%	10	25.6%	0.001
	Complete	224	65.1%	7	77.8%	2	28.6%	17	43.6%	
	Incomplete	80	23.3%	1	11.1%	0	0.0%	12	30.8%	
Twins	0	277	79.8%	8	88.9%	7	100.0%	34	87.2%	0.506
	1	70	20.2%	1	11.1%	0	0.0%	5	12.8%	
Inborn status	0	24	7.0%	0	0.0%	3	42.9%	1	2.6%	0.018
	1	321	93.0%	9	100.0%	4	57.1%	38	97.4%	
Gender	boys	203	58.5%	5	55.6%	4	57.1%	22	56.4%	0.990
	girls	144	41.5%	4	44.4%	3	42.9%	17	43.6%	

A total of 6 infants developed PHH that required surgical intervention. VAD shunting was the preferred temporizing measure due to lower infection rates, with an average duration of 35 days before conversion to VP shunting in 4 children. Serial lumbar punctures were largely avoided due to their limited efficacy and high complication rates. All VAD-treated infants required intermittent CSF tapping, with an average frequency of 2–3 taps per week. Tapping was guided by ventricular measurements and clinical signs of raised intracranial pressure.

At one-year follow-up, 64% of infants showed no significant neurological impairments (Table 7), while 23.2% experienced severe complications, including cerebral palsy or developmental delay. Mortality within the cohort was 20.1%, predominantly in infants with Grade III-IV IVH. Logistic regression analysis confirmed gestational age, corticosteroid treatment, and inborn status as independent predictors of favorable outcomes. Each additional week of gestation reduced the odds of IVH by 26%, while complete corticosteroid administration reduced the odds by 78%. Among the 39 deaths, the majority were attributed to complications of extreme prematurity, including sepsis, necrotizing enterocolitis, and respiratory failure. Only two deaths occurred in infants with Grade IV IVH and progressive PHH, and in these cases, mortality was likely multifactorial, with both systemic and intracranial contributors.

**Table 7:** One-year neurodevelopmental outcomes and surgical interventions among infants with PHH.

Outcome and prognosis			
Variable	Category	Count	%
VP shunt placement	No	114	96.6%
	Yes	4	3.4%
Outcome at 1 year	No complications	105	64.0%
	Paresis	12	7.3%
	Plegia	9	5.5%
	Developmental delay	38	23.2%
Death	No	155	79.9%
	Yes	39	20.1%

## Discussion

The surgical management of intraventricular hemorrhage (IVH) and posthemorrhagic hydrocephalus (PHH) in premature infants remains a complex and evolving challenge in neonatal care. Advances in neonatal intensive care have significantly improved the survival of extremely preterm infants, yet these improvements have also led to an increased prevalence of IVH-related complications, particularly PHH (8). Despite substantial efforts to reduce the incidence of IVH through preventive strategies, large hemorrhages still pose a significant clinical problem, requiring timely and often complex neurosurgical intervention to mitigate long-term neurological damage.

Our study demonstrates that early temporizing measures using ventricular access devices (VAD) followed by permanent ventriculoperitoneal shunt (VP shunt) placement yield favorable outcomes in the management of intraventricular hemorrhage (IVH) and posthemorrhagic hydrocephalus (PHH) in premature infants. Our findings are consistent with recent studies that emphasize the critical role of early intervention and report the need and benefit of initial VAD insertion—with almost all eventually requiring VP shunting—and found that the choice of temporizing technique did not significantly alter the eventual need for a permanent shunt (9, 10). The small number of surgically managed PHH cases in our cohort, although consistent with the known incidence of this complication, limits the generalizability of our conclusions and precludes robust inferential statistics for surgical subgroups. This evidence supports the notion that early CSF diversion, regardless of the specific temporary device used, is essential to stabilize these high-risk infants until they are mature enough for definitive treatment.

In our institution, VAD was preferred over EVD or ventriculosubgaleal shunts as the initial temporizing measure. This preference was based on clinical experience indicating lower infection risk, ease of CSF tapping, and a streamlined workflow for converting to permanent VP shunting. VSG shunts were not employed due to resource constraints and institutional familiarity with VAD-based protocols.

Recent literature has also highlighted promising alternatives that could further optimize outcomes. Emerging techniques such as endoscopic third ventriculostomy with choroid plexus cauterization (ETV/CPC) have shown that in carefully selected patients, nearly one-third may avoid the need for permanent shunting altogether (11). Similarly, neuroendoscopic lavage (NEL), especially when combined with ETV, has demonstrated encouraging results by reducing intraventricular debris and thereby lowering the risk of shunt dependence; one retrospective series noted that a significant proportion of infants managed with NEL did not require permanent shunt placement, with complication rates remaining acceptable (12). Although these advanced methods are still under evaluation, they offer the potential for reducing long-term shunt-related complications, a challenge that persists even with conventional approaches.

The long-term outcomes after VP shunt insertion remain a concern, particularly given that while the time to the first shunt revision is similar across different patient groups, infants with lower gestational ages and more severe IVH tend to require a higher cumulative number of revisions over time (13). This trend underscores the inherent vulnerability of the extremely preterm brain and the enduring impact of severe hemorrhage, even when surgical management is optimized. In this context, the success of temporizing measures such as VADs is reinforced by their ability to bridge the gap until the infant's clinical status allows for more definitive management, without increasing the risk of infection or other complications compared to alternatives like ventriculosubgaleal (VSG) shunts (11).

Moreover, evidence from randomized controlled trials, such as the ELVIS trial, has suggested that earlier intervention is associated with improved two-year outcomes, even though the differences in shunt dependence may not be as pronounced (14). A network meta-analysis further supports the critical importance of timing; it indicated that drainage, irrigation, and fibrinolytic therapy (DRIFT) had an 82% probability of being the most effective strategy in reducing death or severe disability, despite a higher incidence of secondary IVH (13). These findings suggest that while the benefits of DRIFT may be offset by procedural risks, the overall trend favors early, aggressive management to mitigate the long-term neurodevelopmental impact of PHH.

Contemporary guidelines from leading neurological societies also stress the need for individualized, timely treatment protocols. They recommend that treatment strategies be tailored to the specific clinical condition of each infant, taking into account factors such as gestational age, IVH severity, and institutional expertise (15). In our experience, the standardized approach of employing VADs as temporizing devices has been associated with low infection rates and acceptable rates of shunt survival, which is in line with these guidelines. Furthermore, while our protocol did not incorporate techniques like DRIFT or NEL, the accumulating evidence for these methods points to a future where a combination of traditional and innovative strategies may further enhance outcomes by reducing both surgical complications and long-term neurodevelopmental deficits (16, 17). The absence of formal, standardized developmental assessments is a limitation that may affect the precision and comparability of our neurodevelopmental outcomes.

The cumulative evidence from recent studies suggests that although no single temporizing technique has emerged as definitively superior, the common denominator across the literature is the importance of prompt and effective intervention. Early CSF diversion not only stabilizes the infant by alleviating raised intracranial pressure but also provides a critical window during which further interventions can be planned and executed safely. This approach has been shown to yield relatively favorable neurodevelopmental outcomes, with nearly two-thirds of survivors in some cohorts achieving normal developmental milestones at one year (14). However, it is also evident that a significant minority of these infants continue to experience severe impairments, underscoring the need for ongoing research and innovation in this challenging field.

Our findings confirm that early and aggressive surgical management of IVH and PHH is essential to optimize both surgical and neurodevelopmental outcomes in premature infants. While our data support the continued use of VADs as a safe and effective temporizing measure, emerging modalities such as ETV/CPC and neuroendoscopic lavage hold promise for further reducing shunt-related complications and improving long-term cognitive function. These advancements underscore the evolving nature of neonatal neurosurgical care and highlight the critical need for continued research to refine treatment protocols for this vulnerable patient population (18).

## Conclusion

Intraventricular hemorrhage and posthemorrhagic hydrocephalus in premature infants present complex clinical challenges, demanding prompt and tailored management. The long-term outcomes were closely linked to the timing and type of neurosurgical management. Infants managed with VAD demonstrated favorable outcomes, with relatively low infection rates and smooth transitions to permanent shunts. Early intervention combined with consistent follow-up significantly improved survival and developmental prospects, highlighting the importance of preventive strategies and optimal surgical management in this high-risk population.

Clinical outcomes are closely linked to the timing of intervention and the severity of the initial hemorrhage, with temporizing strategies reducing complications and improving the success of subsequent permanent shunting. Standardized monitoring and multidisciplinary coordination between neonatology and neurosurgery are essential to ensuring optimal care and reducing morbidity. This approach enables clinicians to better manage the risks associated with posthemorrhagic hydrocephalus and achieve improved neurodevelopmental outcomes for affected infants.

## Data Availability

The data analyzed in this study is subject to the following licenses/restrictions: the data are not publicly available due to privacy or ethical restrictions. Requests to access these datasets should be directed to david.krahulik@fnol.cz.
